# Processing Effects on Protein Structure and Physicochemical Properties

**DOI:** 10.3390/foods11111607

**Published:** 2022-05-30

**Authors:** Vibeke Orlien, Åsmund Rinnan

**Affiliations:** Department of Food Science, University of Copenhagen, Rolighedsvej 26, DK-1958 Frederiksberg, Denmark; aar@food.ku.dk

## 1. Introduction

Raw materials, whether it is from the animal or plant kingdom, undergo some kind of (domestic or industrial) processing prior to consumption. The exact processing method may influence the protein properties to various extent depending on the processing condition. The precise impact on proteins may be wanted or unwanted depending on the wished product output. Yet, the food industry generally wants, and is depending on, in relation to consumer reliability, the same effect and outcome for every processing batch. It is well-known that different processing techniques will result in different protein changes, and thus also differences in food structures. However, understanding of the underlying mechanisms is still needed. Therefore, it is crucial to evaluate conformational changes of the protein and explore the relationships between protein structure and functional properties during processing by effective detection methods. This Special Issue on Processing Effects on Protein Structure and Physicochemical Properties aims to present the latest knowledge about and developments within analytical methods for detecting and assessing protein structural changes by different modification/processing methods and the relationship with protein functionality and covers a total of 8 articles (6 research papers and 2 reviews). Consequently, this Special Issue of Foods presents insight into coffee bean processing, thermal or freezing processing of fish, the important gluten protein in relation to bread-making and a review concerning how to substitute gluten, the idea of a protein method toolbox, and the importance of using the right statistical analytical tool(s) when deducing knowledge from measured data.

Processing of food raw material is often targeted to specific compounds in order to achieve the desired effect on, e.g., texture and taste. Coffee is an extremely important product and, thereby, also the coffee bean processing. In an extensive and detailed, including development of an MRM method using HPLC-MS/MS for analyzing protein-phenolic modification, study of 14 *Coffea arabica* samples from various processing methods and countries was conducted (all commercially available samples) [1]. The proteins are considered to be the most important compounds for the coffee aroma, hence a good cup of coffee. Free amino nitrogen, free thiol groups, phenolic compound compositions, protein content, browning reactions, molecular weight change, protein digestions, and protein-phenolic modification were measured. It was established that post-harvest treatment affect the protein quality. In conclusion, larger sample set are needed to prove the observed trends and changes. Moreover, the authors conclude that there is a possibility of using the modified peptides as biomarkers, though with more in-depth analysis, in order to bring them closer to coffee quality [1].

Fish is part of a healthy diet due to its content of healthy lipids, but is an easy perishable raw material, and need processing in order to obtain a safe product. Thermal processing will provide both safety and also give the desired sensory features. Fish also contains proteins, and another consequence of heating is structural changes of the proteins leading to a wanted or unwanted fish structure. During heating of fish the proteins will denature and aggregate resulting in harder or firmer texture. However, different proteins denatures at different temperatures. Thus, the aim of this study [2] was to combine a kinetic description of protein denaturation with a rheological description of textural changes. It was found that herring proteins had a denaturation temperature ranging from 31.6 °C to 81.7 °C. Interestingly though, was that that not all transformations occurred according to the same kinetic model. The first two and the last peak are described by 1st order kinetics, while peak 3 is described by 2nd order kinetics. The correlation of temperature-induced molecular (thermal analysis) and macroscopic (rheological measurements) changes is a new and interesting approach. Hence, it was established that changes in the G’ and G” values had a high degree of convergence with denaturing changes in the herring meat. Such knowledge about the thermodynamic properties of raw fish is important for choosing the production method and consequently for the quality of the finished products. The kinetic models developed in this study may be used to predict at which temperature and how fast the denaturation of consecutive protein fractions will take place [2]. Freezing of fish is commonly used to avoid spoilage and deterioration, and to keep freshness and quality during transportation and storage. However, uncontrolled freezing and temperature fluctuations resulting in repeating freezing and thawing cycles causes recrystallization of ice crystals, thereby impacting quality. In the next fish study [3] the effect of repeated freezing–thawing on quality changes of cuttlefish were analyzed in order to provide a theoretical basis for transportation, storage, processing, and marketing in actual production. A huge battery of different analytical methods was used to show that with increasing number of freeze–thaw cycles, the process of ice crystal formation, melting and recrystallization in the muscle cells of cuttlefish caused irreversible physical damage to the muscle cells, leading to a decrease in water holding capacity (WHC), hardness, elasticity and chewiness. Importantly, it was found that the content of TBV-N after the fifth freezing and thawing exceeded the standard of first-class freshness. Thus, it was concluded that the freeze–thaw cycles are very harmful to the quality of the frozen fish, and to keep the temperature stable in the low-temperature food logistics is crucial.

Bread is an everyday food product used worldwide for thousands of years. Therefore, a lot of both empiric and scientific knowledge is established. For example, it is well-known that the gluten protein is a key component in wheat-based breads due to the exceptional functional properties, especially its three-dimensional matrix, which is the basis for the viscoelastic properties, WHC, and gas holding ability. During bread-making heating is the process that causes the desired structural changes of gluten. Therefore, the degree of structural alterations by heat and chemical modifications (ascorbic acid, diacetyl tartaric acid ester, and dithiothreitol) was studied using Fourier transform infrared (FTIR) spectroscopy to elucidate the effect of temperature and additives on the secondary structure of gluten [4]. An increasing content of random coils, α-helices, and β-sheets and a decreasing content of β-turns and antiparallel β-sheets were observed upon heating up to 65 °C with all additives, showing that the secondary structures of gluten transformed into more compact and stable conformation. A chemometric analysis revealed that antiparallel β-sheets were negatively correlated to %creep strain, whereas random coils, α-helices, and β-sheets were not correlated to %creep strain. In conclusion, it was possible to quantitate the overall alteration of gluten polymerization and gluten matrix formation during heating with additive treatments [4]. Weak wheat flours do not provide good bread performance, hence supplementing with gluten can be used in baking industry to improve baking and bread quality. Vital gluten mainly consists of the proteins’ gliadins and glutenins and it is the dynamic interaction between those that enables the formation of a viscoelastic gluten network crucial for a good bread. However, the functional properties of vital gluten may vary considerably between different productions and manufactures. This study [5] investigated the baking performance of 10 vital gluten samples and the dependency of dough water content, since it is still not clear how gluten functionality is determined by its composition, structure and chemical-physical properties in a complex dough system. Based on RP-HPLC, SDS-PAGE and thiol/disulfide analyses it was found that the vital gluten samples had similar gluten content and composition as well the content of free thiols and disulfide bonds, but did not relate to the specific bread volumes. However, different water additions affected bread volumes, thus an optimal specific volume of 1.74–2.38 mL/g (baking mixture) and 4.25–5.49 mL/g (weak wheat flour) was reached for each vital gluten sample depending on its specific water absorption capacity. A molecular mechanism visualized with a schematic presentation of the gluten network formation depending on the water content was given. The authors concluded that the disparities regarding baking performance when using a standardized amount of water was induced by different water absorption capacities [5]. Some people suffer from celiac disease symptoms and cannot tolerate gluten. Therefore, gluten free bread is important, but as shown by the two former studies, gluten is very important for bread structuring. The review on Methods for the Modification and Evaluation of Cereal Proteins for the Substitution of Wheat Gluten in Dough Systems [6] gives the most recent advances published on protein modifications of cereals and pseudocereals to substitute gluten. The analyzed information shows that physical, chemical, enzymatic, or genetic modifications of proteins can in fact improve the final properties of gluten-free baking by the enhancement of interactions with other ingredients. Surely, the effectiveness of treatments depends on the type of cereal protein [6]. The review also presents different methodologies for measuring the gluten substitution and the related gluten-free dough properties.

To end our story on processing effects on protein structure and physicochemical properties, the concept of a protein method toolbox and the importance of using the right statistical analytical tools is presented in a review [7] and a highlighted communication [8]. The review provides a tangible overview of the methods currently available for determining protein functionality and related molecular characteristics. The methods of functionality include solubility, water holding capacity, oil holding capacity, emulsion property, foam property, and gelation, the molecular characteristic methods include electrophoresis, surface hydrophobicity and charge, molecular interaction, and thermal property, and the spectroscopic methods include ultraviolet-visible, Fourier transform infrared, Raman, circular dichroism, fluorescence and nuclear magnetic resonance. First and foremost, there is a need to establish a standardization of the analytical methods for assessing functional properties in order to compare and understand results from different investigations. We suggest that a protein analytical toolbox that couples different chemical, physical and spectroscopic analyses in relation to protein functionality and molecular characterization will improve the rational design of different protein ingredients [7]. As an example, the importance of using two statistical analytical tools, PCA and Pearson’s correlation, for a large set of data obtained from fava bean concentrates processed by different process conditions [8] supports the need of such a toolbox. In this study [8], fava bean concentrates were processed at various pH (2, 4, 6.4 and 11), temperature (55, 75 and 95 °C) and treatment duration (30 and 360 min) to obtain different ingredients that were tested at two conditions (pH 4 and 7) for their foam and emulsion properties and correlated to their molecular characteristics (charge, solubility and intrinsic fluorescence). Generally, the two beverage functionalities, foam and emulsion properties, were not correlated to each other. This finding was explained as a consequence of association with different protein and non-protein attributes as fava bean concentrate is a multi-component matrix. The overall importance of this paper is that the results from the two statistical tools are not fully comparable but do complement each other. Pearson’s correlation validated the associations between functionalities and physico-chemical properties, and PCA suggested the impact of process conditions on ingredient properties along with some obvious associations between the properties.
